# Prevalence of hepatitis B and C, HIV, and syphilis among people who inject drugs in Germany

**DOI:** 10.1093/eurpub/ckac131.200

**Published:** 2022-10-25

**Authors:** G Steffen, A Krings, R Zimmermann

**Affiliations:** Department of Infectious Disease Epidemiology, Robert Koch Institute, Berlin, Germany

## Abstract

**Background:**

We piloted a future monitoring system among people who inject drugs (PWID) in Germany (DRUCK2.0). Monitoring is needed to inform and support the viral hepatitis/HIV/STI elimination process in Germany by providing regular up-to-date prevalence and behavioural data for this key population.

**Methods:**

PWID aged 16+ years who injected drugs within the last 12 months were recruited by time location sampling via low threshold drug services and opioid substitution treatment (OST) practices during routine services in Berlin and Bavaria between 01/06/2021 and 28/02/2022. All participants filled a questionnaire on sociodemographics, behaviour and access to care and were tested for hepatitis B and C (HBV, HCV), HIV and syphilis using capillary dried blood spots. All received a 10 Euro incentive voucher.

**Results:**

In total, 495 PWID were included, median age was 39 years [range 18-66], 68% (336/494) were male, and 23% (114/492) born outside Germany, mostly in eastern Europe. Of all participants, 58% (275/477) reported recent use of unsafe needles/syringes and 77% (372/485) detention experience. Current OST was reported by 62% (304/487). Prevalence was 46% (229/495) for cured HCV, 26% (130/495) for active HCV, 17% (80/482) for cured HBV, 1.2% (6/483) for active HBV, 2.7% (13/482) for HIV and 2.1% (10/473) for previous Syphilis. Serological HBV vaccination coverage was 24% (115/475). Of all, 95% (453/475) reported previous HCV testing. Of those with cured/active HCV infection 88% (296/337) knew about their infection and 56% (161/285) of them reported previous/current treatment.

**Conclusions:**

To decrease the heavy burden of infection among PWID in Germany, targeted measures regarding access to HCV treatment, HBV vaccination, and harm reduction (safer use measures, OST) need to be implemented and rolled out. Nationwide regular monitoring of indicators in this key population is needed to guide the elimination progress of viral hepatitis and HIV in Germany.

**Key messages:**

• High burden of active Hepatitis C infection and low Hepatitis B vaccination coverage among people who inject drugs in Berlin and Bavaria require improved access to treatment and prevention efforts.

• National Monitoring is needed to inform and support the viral hepatitis/HIV/STI elimination process among people who inject drugs in Germany.

